# (HIIT-The Track) High-Intensity Interval Training for People with Parkinson’s Disease: Individual Response Patterns of (Non-)Motor Symptoms and Blood-Based Biomarkers—A Crossover Single-Case Experimental Design

**DOI:** 10.3390/brainsci13060849

**Published:** 2023-05-24

**Authors:** Elvira S. Amaral Gomes, Odile A. Van den Heuvel, Marc B. Rietberg, Vincent De Groot, Mark A. Hirsch, Wilma D. J. Van de Berg, Richard T. Jaspers, Chris Vriend, Tim Vanbellingen, Erwin E. H. Van Wegen

**Affiliations:** 1 Department of Rehabilitation Medicine, Amsterdam UMC, Location Vrije Universiteit Amsterdam, 1081 HV Amsterdam, The Netherlands; e.s.amaralgomes@amsterdamumc.nl (E.S.A.G.); v.degroot@amsterdamumc.nl (V.D.G.); 2 Amsterdam Movement Sciences, Rehabilitation & Development, Amsterdam UMC, Location Vrije Universiteit Amsterdam, 1081 HZ Amsterdam, The Netherlands; 3 Department of Anatomy & Neurosciences, Amsterdam UMC Location Vrije Universiteit Amsterdam, 1081 HV Amsterdam, The Netherlandsc.vriend@amsterdamumc.nl (C.V.); 4 Department of Psychiatry, Amsterdam UMC, Location Vrije Universiteit Amsterdam, 1081 HV Amsterdam, The Netherlands; 5 Amsterdam Neuroscience, Compulsivity, Impulsivity & Attention, Neurodegeneration, Amsterdam UMC, Location Vrije Universiteit Amsterdam, 1081 HZ Amsterdam, The Netherlands; 6 Amsterdam Neuroscience, Neuroinfection & Neuroinflammation, Amsterdam UMC, Location Vrije Universiteit Amsterdam, 1081 HZ Amsterdam, The Netherlands; 7 Carolinas Medical Center, Atrium Health Carolinas Rehabilitation, Department of Physical Medicine and Rehabilitation, Charlotte, NC 28203, USA; 8 Wake Forest School of Medicine, Department of Orthopaedic Surgery and Rehabilitation, Winston-Salem, NC 27157, USA; 9 Amsterdam Neuroscience, Neurodegeneration, Amsterdam UMC, Location Vrije Universiteit Amsterdam, 1081 HZ Amsterdam, The Netherlands; 10 Laboratory of Myology, Department of Human Movement Science, Faculty of Behavioural and Movement Sciences, Location Vrije Universiteit Amsterdam, 1081 BT Amsterdam, The Netherlands; 11 Amsterdam Movement Sciences, Rehabilitation & Development, Tissue Function & Regeneration, Amsterdam UMC, Location Vrije Universiteit Amsterdam, 1081 HZ Amsterdam, The Netherlands; 12 Gerontechnology and Rehabilitation Group, University of Bern, 3008 Bern, Switzerland; 13 Neurocenter, Luzerner Kantonsspital, 6000 Lucerne, Switzerland; 14 Amsterdam Movement Sciences, Ageing & Vitality, Amsterdam UMC, Location Vrije Universiteit Amsterdam, 1081 HZ Amsterdam, The Netherlands; 15 Amsterdam Neuroscience, Neurovascular Disorders, Amsterdam UMC, Location University of Amsterdam, 1105 AZ Amsterdam, The Netherlands

**Keywords:** Parkinson’s disease, rehabilitation, exercise, high intensity interval training, endurance training, neuroprotection, brain-derived neurotrophic factor, neurofilament proteins

## Abstract

Introduction: Physical exercise is receiving increasing interest as an augmentative non-pharmacological intervention in Parkinson’s disease (PD). This pilot study primarily aimed to quantify individual response patterns of motor symptoms to alternating exercise modalities, along with non-motor functioning and blood biomarkers of neuroplasticity and neurodegeneration. Materials & Methods: People with PD performed high-intensity interval training (HIIT) and continuous aerobic exercise (CAE) using a crossover single-case experimental design. A repeated assessment of outcome measures was conducted. The trajectories of outcome measures were visualized in time series plots and interpreted relative to the minimal clinically important difference (MCID) and smallest detectable change (SDC) or as a change in the positive or negative direction using trend lines. Results: Data of three participants were analyzed and engaging in physical exercise seemed beneficial for reducing motor symptoms. Participant 1 demonstrated improvement in motor function, independent of exercise modality; while for participant 2, such a clinically relevant (positive) change in motor function was only observed in response to CAE. Participant 3 showed improved motor function after HIIT, but no comparison could be made with CAE because of drop-out. Heterogeneous responses on secondary outcome measures were found, not only between exercise modalities but also among participants. Conclusion: Though this study underpins the positive impact of physical exercise in the management of PD, large variability in individual response patterns to the interventions among participants makes it difficult to identify clear exercise-induced adaptations in functioning and blood biomarkers. Further research is needed to overcome methodological challenges in measuring individual response patterns.

## 1. Introduction

Parkinson’s disease (PD) is a highly disabling progressive neurodegenerative disorder, characterized by degeneration of multiple neurotransmitter systems, most notably the dopaminergic system [1]. While traditionally referred to as movement disorder, there is growing awareness of burdensome non-motor symptoms such as cognitive decline, anxiety and depression, and disturbed sleep [2]. As there is no cure for PD, pharmacological treatments are available for symptom relief; however, several motor and non-motor symptoms respond insufficiently to medication, and daily functioning is increasingly limited by adverse drug effects [3]. Considering the gradual worsening of symptoms and subsequent threat to participation in everyday activities, independent living, and quality of life, there is a need for additional non-pharmacological interventions to alleviate the disease burden and preferably modify progression.

Engaging in physical exercise is acknowledged as an augmentative and safe therapy for people with PD [4]. Whereas continuous aerobic exercise (CAE) is commonly used in clinical settings, training sessions are considered time-consuming [5]. A relatively new, yet promising, exercise modality in rehabilitation practice is high-intensity interval training (HIIT), characterized by repeated intervals of high-intensity activity bouts interspersed with active rest at low intensity. HIIT induces similar, or even superior, cardiovascular responses compared to CAE, in a significantly shorter training time [6]. This increased efficiency is expected to enhance motivation as well as adherence to exercise programs and stimulate a more active lifestyle. While HIIT has been revealed to be feasible for people with PD [7], the number of studies exploring its effect on motor and non-motor symptoms, and particularly in comparison to CAE, are scarce.

Fernandes et al. [8] performed a pilot randomized controlled single-blind trial to study the effect of 12 weeks of HIIT vs. moderate-intensity continuous exercise (MICE) training in PD. They enrolled 20 participants, with Hoehn–Yahr stage 1–3, who performed walking/jogging training three times per week for 12 weeks. The HIIT protocol consisted of a 1-min walking/jogging bout at a rating on a perceived exertion (Borg scale RPE) scale 15–17 level alternating with 2 min of walking at 9–11 level of RPE during a 25-min session. MICE training consisted of 26 min of walking/jogging at 11–14 level of RPE. HIIT improved 6-min walk test distance and increased endothelial reactivity (a marker for increased blood flow). These values did not change with MICE. They did not measure non-motor outcomes.

Animal models and human studies reveal exercise-induced neuroprotective changes at the molecular level, with great interest in brain-derived neurotrophic factor (BDNF) [9,10]. The involvement in neuronal maturation, survival, and repair renders BDNF as a candidate biomarker for monitoring treatment response [11]. The role of exercise intensity on serum BDNF concentration was studied in an RCT (n = 30) and results indicated significantly increased BDNF levels from baseline to post-intervention, only in response to HIIT, possibly caused by HIIT-induced brain hypoxia [12] or increased neurotrophic expression in skeletal muscle [13]. Another relevant biomarker to monitor treatment response in PD is neurofilament light (NfL), a structural protein filling the axonal cytoplasm. Upon neuroaxonal damage, it is released into the extracellular fluid [14] leading to elevated serum NfL levels in people with PD compared to age-matched healthy controls [15].

Despite promising findings on physical exercise in the management of PD, there is a large variety of intervention types and doses. The heterogeneous disease course is an additional confounding factor to work toward optimal treatment strategies. Gaining insight into individual response patterns of, primarily, motor and non-motor symptoms as well as the stability and responsiveness of blood biomarkers of neuroplasticity and neurodegeneration to alternating exercise modalities (HIIT and CAE) using a crossover single-case experimental design (SCED) [16] was, therefore, the aim of this study. Elucidating these patterns is valuable in the preparation of a well-powered RCT to, ultimately, develop appropriate exercise interventions which adequately alleviate disease burden.

## 2. Materials & Methods

The study protocol was published in van Wegen et al., 2020 [16].

### 2.1. Study Design

Individual response patterns of motor and non-motor symptoms, as well as serum blood biomarkers of neuroplasticity (BDNF) and neurodegeneration (NfL) were quantified using a crossover SCED (A_1_-B-A_2_-C-A_3_ or A_1_-C-A_2_-B-A_3_), which enabled participants to participate in the HIIT (B-phase) and CAE (C-phase) intervention. During baseline, wash-out, and follow-up, participants did not receive additional exercise training. Intervention order was randomized and repeated assessment of outcome measures was conducted (Supplementary Figure S1: timeline and assessment schedule).

### 2.2. Study Population

Four individuals with idiopathic PD were recruited from the outpatient clinic of Amsterdam UMC location Vrije Universiteit medical center (VUmc), Amsterdam, the Netherlands. Participants were asked to maintain a stable medication regime in the 4 weeks prior to and during study enrollment unless adaptations were medically necessary. This study was approved by the Medical Ethics Committee of VUmc (NL78096.029.21) and conducted in accordance with the Declaration of Helsinki. Participants provided written informed consent at intake.

### 2.3. Eligibility Criteria

Inclusion criteria: (1) diagnosis of PD, based on the UK Brain Bank criteria [17]; (2) Hoehn and Yahr (H&Y) stage < 4; (3) sufficient cognitive ability to understand training instructions [Montreal Cognitive Assessment (MoCA) score > 21 points]; (4) age between 55 and 80 years; 5. able to provide written consent. Exclusion criteria: (1) history of neurological deficits other than PD; (2) severe response fluctuations to dopaminergic medication, according to the treating neurologist; (3) cardiovascular disorder or cardiac risk prohibiting participation in intensive physical exercise [Lausanne Protocol: > 2 items scored ‘yes’ (questionnaire)] [18]; (4) psychiatric, musculoskeletal, or metabolic disorder prohibiting participation in intensive physical exercise; 5. enrolled in a supervised exercise program 2 months prior to inclusion.

#### Training Protocol

Subjects performed HIIT or moderate CAE on a stationary bicycle (Lode Corival, Lode Inc., Groningen, The Netherlands) 3×/week for two periods of 4 weeks in a group of four subjects. The order of the intervention phases will be randomized. Training took place on Mondays, Wednesdays, and Fridays, to allow for sufficient recovery periods between training sessions. Each HIIT session was about 30 min of interval training, starting with a 5 min of warm-up at 25–35% W-max. Subsequently, six to eight blocks of HIIT were performed, alternating 45 s at >85% VO2-max, with 90 s at 30–40% W-max. After the interval block, 5 min of cooling-down was performed at 20–35% Wmax. The workload was progressively adapted using a fixed weekly schedule or upon therapist decision by adding intervals at the end of the sessions. Each CAE session was about 50 min of continuous training at a preset workload of about 55% W-max. Workload was progressively adapted after every week by reducing rest time, based on a fixed schedule or upon therapist decision. A trained physiotherapist–supervisor, assisted by GCP-trained interns from the Faculty of Human Movement Sciences, assisted the subjects with water, music, and motivational encouragement. Throughout the study, patients were asked to maintain their regular medication. The Ecological Momentary Assessments were taken weekly (A phases) or every other day (B/C phases) using the online survey throughout the study period acted automatically as reminders and helped with compliance with training and assessments.

### 2.4. Outcome Measures

Timeline and assessment schedule for demographics, clinical characteristics, and all outcome measures (disease severity, disease status, cognitive function, activities of daily living, mood, quality of life, sleep quality, motor capacity, daily mood, cognitive function, and sleep quality, physical (in)activity, blood biomarkers of neuroplasticity, and neurodegeneration) are presented elsewhere [16]. In total, 6 outcome measures (motor symptoms, disease status, cognitive function, mood, blood biomarkers of neuroplasticity, and neurodegeneration) are presented here. Data on the other outcome measures can be found in Supplementary Tables S1–S3. Assessments were performed in ‘on’-state, preferably 1 to 1.5 h after medication intake.

### 2.5. Primary Outcome Measure: Motor Symptoms

Motor symptoms were assessed using the Movement Disorder Society (MDS) sponsored revision of the Unified Parkinson’s Disease Rating Scale (MDS-UPDRS) part III. Total UPDRS III scores ranged between 0 (minimum motor disability) and 132 (maximum motor disability) points [19]. The minimal clinically important difference (MCID) in the UPDRS III score for improvement was a decrease of 3.25 points, while an increase of 4.62 points indicated a worsening of motor function [20].

### 2.6. Secondary Outcome Measures

#### 2.6.1. Disease Status

Disease status was assessed by the UPDRS part II for motor experiences of daily living. Total UPDRS II scores ranged between 0 (no disability) and 52 (severe disability) points [19] with a decrease of 3.05 points and an increase of 2.51 points as the MCID for improvement and worsening in motor experiences of daily living, respectively [21].

#### 2.6.2. Cognitive Function

Cognitive function was assessed using the Parkinson’s Disease Cognitive Functional Rating Scale (PD-CFRS) with a total score range from 0 to 24 points. Higher scores indicated greater functional disability due to cognitive impairment [22]. The MCID in PD-CFRS score for improvement and worsening is 2 points [23].

#### 2.6.3. Mood

Mood was assessed using the Beck Depression Inventory (BDI). Total BDI score ranged from 0 (no depression) to 63 (severe depression) points. A decline of 13.8% from baseline in BDI score was considered the smallest detectable change (SDC) towards improvement [24]. The MCID in BDI score has, to our knowledge, not been reported in people with PD.

#### 2.6.4. Biomarkers of Neuroplasticity and Neurodegeneration in Blood

Blood samples for serum BDNF and NfL analysis were collected once a week during baseline and interventions and, to reduce patient burden, once every two weeks during wash-out and follow-up. Blood samples were collected from the antecubital vein using anticoagulant-free tubes (3 × 20 mL) (directly before training sessions in the intervention periods and directly before assessments in the baseline, wash-out, and follow-up periods). Serum BDNF and NfL levels were measured using a commercially available in-house validated kit (Quanterix Simoa, Quanterix, Billerica, MA, USA) [15] and an in-house developed Homebrew Simoa assay [25], respectively, on the Simoa^TM^ HD-1 Analyzer (Quanterix, Billerica, MA, USA) according to the manufacturer’s instructions. Samples were measured in duplicate (mean coefficient of variation from duplicate sample concentrations of 3.64% (range: 0.06 to 11.29).

## 3. Data Analysis

We used visual inspection to explore individual response patterns within and between study phases for each participant separately, supported with statistics. Data are presented in time series plots using R (version 4.0.3) [26]. The trajectories of outcome measures that were assessed before and after each study phase are interpreted relative to the MCID (dashed line) and SDC (triangle), which were superimposed onto the graphs. The MCID indicates the smallest change (improvement or worsening) considered important to the patient, whereas, the SDC represents the amount of variation in the assessment tools [27]. If the SDC is not reported, it was calculated as standard error of measurement (SEM) * 1.96 * √2 [28]. The SEM was obtained using standard deviation of measure * √ (1 − reliability coefficient) [27]. Time series plots of weekly assessed outcome measures are provided with trend lines (dotted line), expressed as regression coefficients (β). The trajectories of these weekly assessed outcome measures are interpreted as changes in the positive or negative direction. Longitudinal regression analysis was used to assess whether these changes were significantly different between study phases. The level of significance was set at *p* < 0.05.

## 4. Results

**Figure 1:**
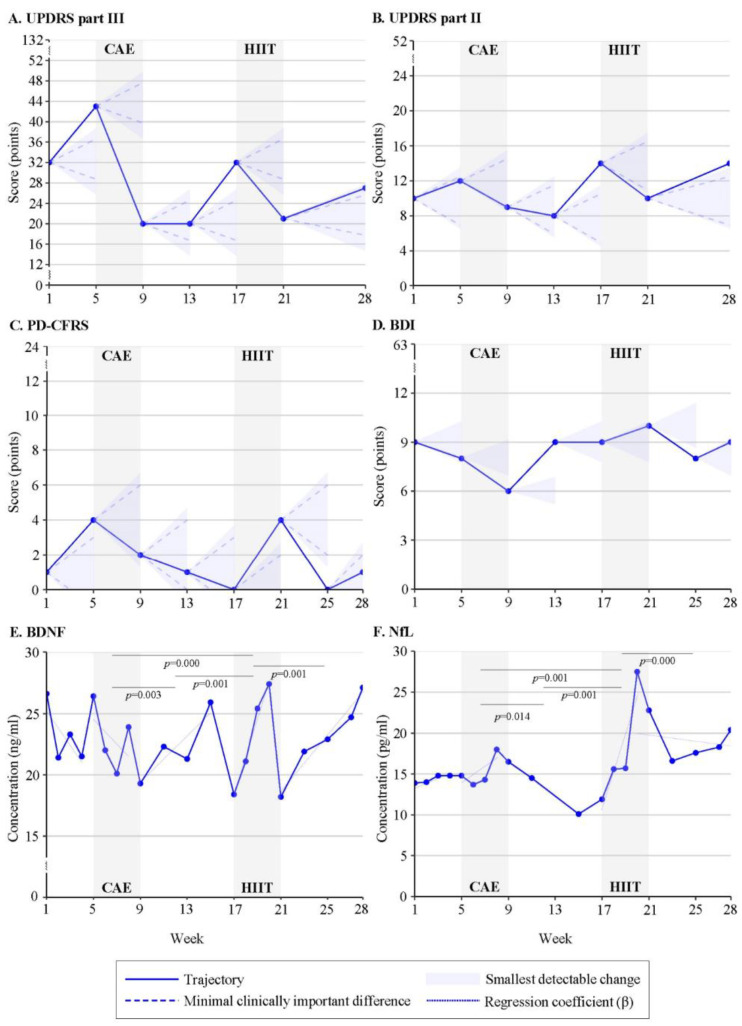
Response patterns to Continuous Aerobic Exercise (CAE) and High-Intensity Interval Training (HIIT) of participant 1 for (**A**) UPDRSIII = Unified Parkinson’s Disease Rating Scale: motor examination, (**B**) UPDRSII = Unified Parkinson’s Disease Rating Scale: motor experiences of daily living, (**C**) PD-CFRS = Parkinson’s Disease Cognitive Functional Rating Scale, (**D**) BDI = Beck Depression Inventory, (**E**) BDNF = Brain-Derived Neurotrophic Factor, (**F**) NfL = Neurofilament Light.

**Figure 2:**
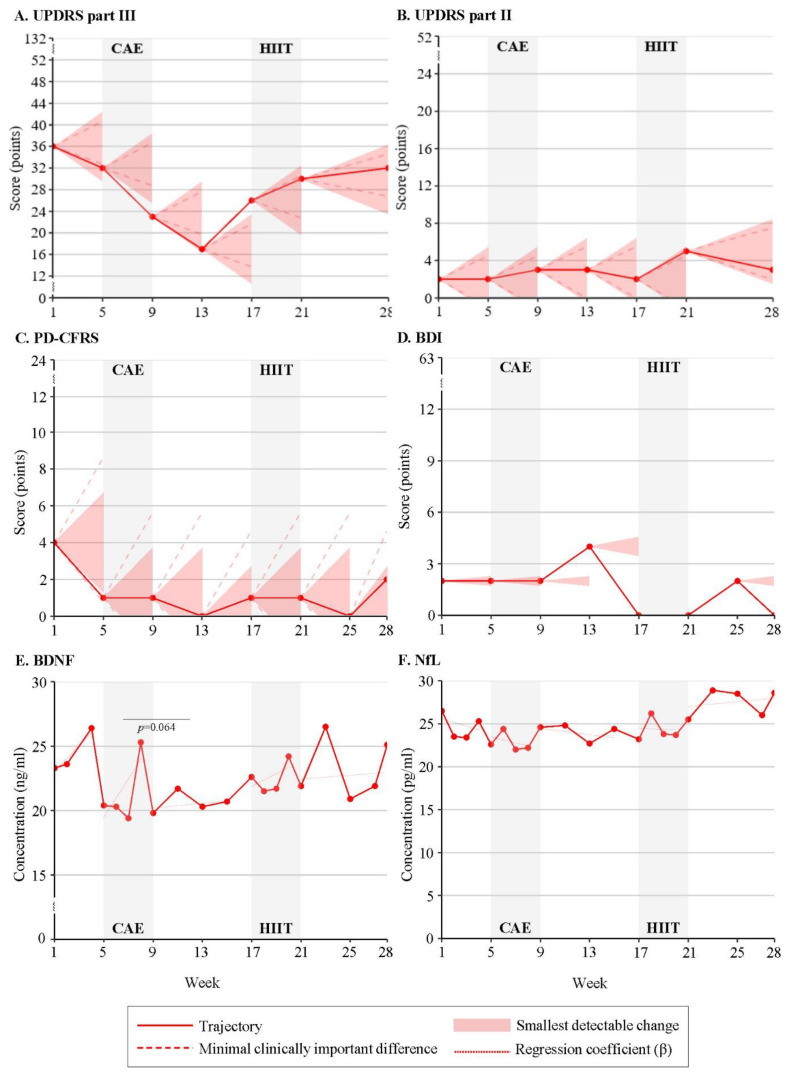
Response patterns to Continuous Aerobic Exercise (CAE) and High-Intensity Interval Training (HIIT) of participant 2 for (**A**) UPDRSIII = Unified Parkinson’s Disease Rating Scale: motor examination, (**B**) UPDRSII = Unified Parkinson’s Disease Rating Scale: motor experiences of daily living, (**C**) PD-CFRS = Parkinson’s Disease Cognitive Functional Rating Scale, (**D**) BDI = Beck Depression Inventory, (**E**) BDNF = Brain-Derived Neurotrophic Factor, (**F**) NfL = Neurofilament Light.

**Figure 3:**
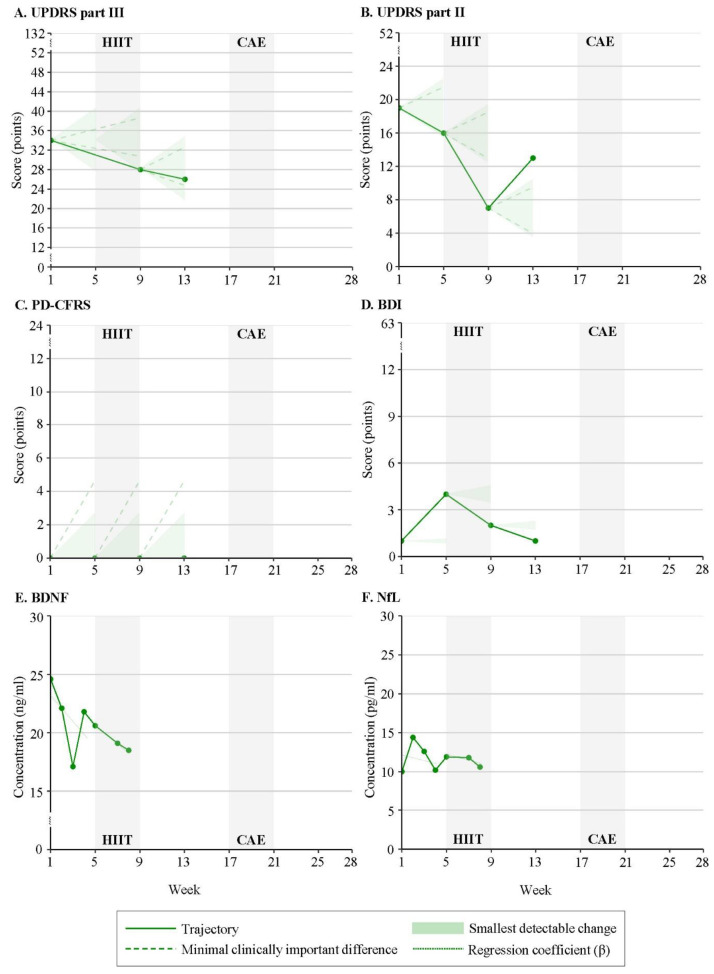
Response patterns to High-Intensity Interval Training (HIIT) and Continuous Aerobic Exercise (CAE) of participant 3 for (**A**) UPDRSIII = Unified Parkinson’s Disease Rating Scale: motor examination, (**B**) UPDRSII = Unified Parkinson’s Disease Rating Scale: motor experiences of daily living, (**C**) PD-CFRS = Parkinson’s Disease Cognitive Functional Rating Scale, (**D**) BDI = Beck Depression Inventory, (**E**) BDNF = Brain-Derived Neurotrophic Factor, (**F**) NfL = Neurofilament Light.

Table 1 outlines demographics and clinical characteristics assessed at baseline. One of the four enrolled participants withdrew from the study in week 1 because of personal circumstances. Participants 1 and 2 were exposed to CAE in the first intervention period, while participant 3 started with HIIT and dropped out halfway through the wash-out (week 13) because of self-induced changes in medication due to improved functioning and non-compliance. Time series plots of individual response patterns are presented in Figure 1, Figure 2 and Figure 3 (Supplementary Table S4: summary in words).

### 4.1. Primary Outcome Measure: Motor Symptoms 

Participants 1 and 2 consistently demonstrated improvement in motor function (UPDRS III) in response to the CAE intervention which initially stabilized into subsequent wash-out for participant 1 (Figure 1A), while the UPDRS III score continued to decrease for participant 2 (Figure 2A). Both participants presented worsening motor function from week 4 of the wash-out phase. Improved motor function in response to HIIT was observed for participants 1 and 3 (Figure 3A).

### 4.2. Secondary Outcome Measures

#### 4.2.1. Disease Status: Motor Experiences of Daily Living

Improvement in independence (UPDRS II) in response to the intervention(s) was identified for participants 1 (Figure 1B) and 3 (Figure 3B), with worsening in subsequent wash-out phases. Participant 2 presented an opposite trajectory of UPDRS II score (worsening of independence) during HIIT (Figure 2B).

#### 4.2.2. Cognitive Function

Participant 1 showed opposite responses to the interventions, with a decrease in PD-CFRS score (cognitive improvement) during CAE and an increase in PD-CFRS score (cognitive worsening) in response to HIIT (Figure 1C). No clinically relevant changes in cognitive function were observed for participant 2 (Figure 2C) and participant 3 (Figure 3C) maintained a score of zero during the study.

#### 4.2.3. Mood

Decreases in BDI score, which exceeded the SDC for improvement in mood, were identified for participants 1 (Figure 1D) and 3 (Figure 3D), with clear differences between exercise modalities. HIIT induced a decrease in BDI score for participant 3, while such a positive change in BDI score was only observed for participant 1 in response to CAE. Opposite trajectories in BDI score were also found in periods without additional exercise training. Participant 2 showed no changes in BDI score in response to the interventions, with a score of zero (no depressive symptoms) at the start of HIIT (Figure 2D).

### 4.3. Biomarkers of Neuroplasticity and Neurodegeneration in Blood

#### 4.3.1. Brain-Derived Neurotrophic Factor (BDNF)

Participants 1 (Figure 1E) and 2 (Figure 2E) demonstrated an increase in BDNF concentration in response to HIIT (β = 0.5 and β = 3.13, respectively); whereas a decrease in BDNF concentration was seen during CAE (participant 1: β = −0.94; participant 2: β = 1.38). For participant 1, the direction of change in BDNF concentration was significantly different between the exercise modalities (*p* = 0.000). Both participants consistently revealed an increase in BDNF concentration during the wash-out and follow-up phases. In contrast, a decrease in BDNF concentration after HIIT was observed for participant 3 (β = −0.71) (Figure 3E).

#### 4.3.2. Neurofilament Light (NfL)

For participant 1, the increases in NfL concentration during CAE (β = 1.02) and HIIT (β = 4.69) were significantly different (*p* = 0.001) and followed by a decrease in NfL concentration in the subsequent wash-out and follow-up (Figure 1F). Participant 2 revealed similar, yet reverse, patterns, with an increase in NfL concentration in response to CAE (β = −0.36) and HIIT (β = −0.09) (Figure 2F). Changes in NfL concentration in periods without additional exercise training were in the opposite direction (CAE: β = −0.14; HIIT: β = 0.17). Similar to participant 1, a decrease in NfL concentration in response to HIIT was observed for participant 3 (β = −0.38) (Figure 3F).

## 5. Discussion

Using crossover SCED, we primarily aimed to gain insight into individual response patterns of motor symptoms, alongside non-motor functioning and the stability, as well as responsiveness, of serum blood biomarkers of neuroplasticity and neurodegeneration to alternating exercise modalities in people with PD. Engaging in physical exercise was demonstrated to be effective for reducing motor symptoms independent of exercise modality for participant 1, while participant 2 only showed a clinically relevant improvement in motor function after CAE. The direction and size of change on secondary outcome measures revealed variability not only between exercise modalities but also among participants. While participant 1 appeared to benefit most from the CAE intervention, change scores in secondary outcome measures for participant 2 often did not exceed the MCID for improvement or worsening. Participant 3 demonstrated improvement in motor and non-motor functioning, but solely participated in HIIT so no comparison between exercise modalities could be made. Changes in biomarker concentrations during the interventions additionally varied among participants.

An improvement in motor symptoms, independent of exercise modality, is in line with our expectations and underscores the positive impact of physical exercise in the management of PD [4]. For participant 1, the decrease in UPDRS III score was visually larger in response to CAE when compared to HIIT. However, a clinically relevant decrease in UPDRS III score was still achieved during HIIT and achieved within a shorter training duration (CAE: 600 min; HIIT: 360 min). In previous studies, where improvements in motor symptoms were found, participants were exposed to longer intervention periods (minimally 7 weeks) and higher training volume [29]. Although change scores on secondary outcome measures for participant 2 were often smaller than the MCID, it is plausible that exercise-induced absence of symptom worsening is of added value considering the progressive disease course.

Both the SDC and MCID are important benchmarks for interpreting change scores on outcome measures. If the SDC is smaller than the MCID, a clinically relevant change can be distinguished from measurement error [27]. The majority of outcome measures had an SDC that was slightly larger than the MCID, which might be due to data not being obtained from the same population. Our findings on secondary outcome measures should therefore be interpreted with caution. Consequently, for change scores as large as the MCID, we cannot be certain that observed changes were not due to measurement error. However, observed differences between these reference points are relatively small and this study is the first to examine individual response patterns of (non-)motor symptoms and blood biomarker concentrations in response to exercise modalities.

Because participants were physically active before enrolling in this study, the influence of exercise and subsequent comparison between HIIT and CAE on motor and, in particular, non-motor symptoms might have been masked. At baseline, only participant 2 scored above the cut-off for functional disability due to cognitive impairment [23]. The possibility of a ceiling effect is also suggested for mood as none of the participants reported depressive symptoms at baseline and all had a BDI score below the cut-off for a positive screen for depression [30]. Future studies investigating the effect of physical exercise on (but not limited to) mood should consider this; for example, by using a cut-off score for inclusion. Information on fatigue in response to physical exercise is additionally important as it might be helpful to explain heterogeneity in outcome measures.

Minimally invasive measures for monitoring disease severity and treatment response have attracted interest to advance knowledge of possible mechanisms underlying the health-promoting effects of physical exercise in PD. Blood biomarker concentrations varied between participants and, interestingly, participant 2 showed favorable changes in BDNF and a small decrease in NfL concentration, independent of the exercise modality. Participant 1 demonstrated a delayed response in the concentration of NfL after both interventions, with favorable changes only identified in subsequent wash-out phases. Conversely, participant 2 showed positive and acute changes in NfL concentration in response to CAE and HIIT. It can be hypothesized that disease duration or stage and age alter the influence of physical exercise on NfL. However, these measures are shown to fluctuate heavily, which could also reflect biological variability during the day or weeks, and to date, there is little known about serum BDNF and NfL fluctuations in time.

Even though this study is the first to shed light on individual response patterns of motor and non-motor symptoms as well as blood biomarkers of neuroplasticity and neurodegeneration to alternating exercise modalities in people with PD, caution is warranted when interpreting our findings. There is large variation in participant responses to the interventions and on outcome measures, which hampers finding clear patterns but provides awareness of important within-subject changes over time, also in response to interventions. However, we specifically aimed to quantify the patterns of longitudinal variation and our results suggest that only performing pre-post measurements in clinical trials may mask these variable patterns. The within-subject variation as well as the between-subject differences in responses may be a reason why small trials with just pre-post assessments of outcomes often produce neutral or negative results. The inclusion of only participants with mild to moderate PD who were relatively active might underestimate the influences of HIIT and CAE because engaging in physical exercise might have a greater impact on motor and non-motor functioning in more advanced disease stages or in a sedentary population. Additionally, maximum exercise capacity was measured prior to the first intervention period, so the training load was not adjusted while participants might have improved. One may notice that CAE and HIIT require different training times, preventing direct comparison due to different treatment dosages. Our hypothesis was that HIIT is a more efficient intervention (i.e., the same or more effects in less training time) which is why we did not align intervention durations. It can also be questioned whether intervention periods were long enough for exercise-induced changes to emerge, given the advice from a recent systematic review about aerobic training (minimally 8 weeks) [29]. As HIIT is relatively new within rehabilitation practice, there is not much documented on the effect on motor and, in particular, non-motor symptoms in PD. Both exercise modalities are therefore incorporated in the follow-up RCT, with an extended intervention period of 8 weeks and a primary focus on anxiety and depressive symptoms (ClinicalTrials.gov: NCT05357638).

## 6. Conclusions

Physical exercise was demonstrated to be beneficial to maintain or improve motor and non-motor functioning. Variability in individual response patterns in combination with treatment-unrelated fluctuations in outcome measures hindered finding clear exercise-induced adaptations. Research on individual response patterns of motor and non-motor symptoms should therefore be continued to identify reliable measures for monitoring disease and responses to interventions. Future work ought to focus on which personal or environmental factors might contribute to different participant responses between exercise modalities (e.g., phenotype, disease severity, duration of speed of progression, apathy, depression, co-morbidities, caregiver support, etc.) and additionally, whether one exercise modality is superior to the other.

## Figures and Tables

**Table 1:** Demographics and clinical characteristics at baseline.

	Study Population		
	Participant 1	Participant 2	Participant 3
Age, years	56	75	58
Sex, F/M	F	M	M
BMI	22.58	25.03	22.74
Disease duration, years	14.25	2	13.50
H&Y stage	2	3	3
LEDD total, mg	798	450	500
UPDRS I score, points	12	9	1
UPDRS III score, points	32	36	34
UPDRS IV score, points	8	1	8
Cognition (MoCA), points	29	24	27

F = Female, M = Male, BMI = Body Mass Index, H&Y = Hoehn and Yahr stage, LEDD = Levodopa Equivalent Daily Dose [28], UPDRS = Unified Parkinson’s Disease rating Scale (UPDRS I: mentation, behavior, mood; UPDRS IV: complications of therapy), MoCA = Montreal Cognitive Assessment.

## Data Availability

Data available upon request.

## Supplementary Materials

The following supporting information can be downloaded at: https://www.mdpi.com/article/10.3390/brainsci13060849/s1, Figure S1: Timeline and assessment schedule using a crossover single-case experimental design with alternating treatment conditions, i.e., exercise modalities; Table S1: Change scores (absolute values or beta (β)) on secondary outcome measures of participant 1; Table S2: Change scores (absolute values or beta (β)) on secondary outcome measures of participant 2; Table S3: Change scores (absolute values or beta (β)) on secondary outcome measures of participant 3; Table S4: Changes (improvement or worsening) on outcome measures according to the smallest detectable change, per study phase
