# Supplementary material for: (HIIT-The Track) High-Intensity Interval Training for People with Parkinson’s Disease: Individual Response Patterns of (Non-)Motor Symptoms and Blood-Based Biomarkers—A Crossover Single-Case Experimental Design
Source: Brain Sci. 2023 May 24;13(6):849. doi: 10.3390/brainsci13060849 (PMC10296509; doi:10.3390/brainsci13060849)
Supplement: Supplementary file 1 [file brainsci-13-00849-s001.zip › brainsci-2393754-supplementary.pdf]

Supplementary Figure S1 Timeline and assessment schedule using a crossover single-case experimental design with alternating treatment conditions, i.e., exercise modalities.

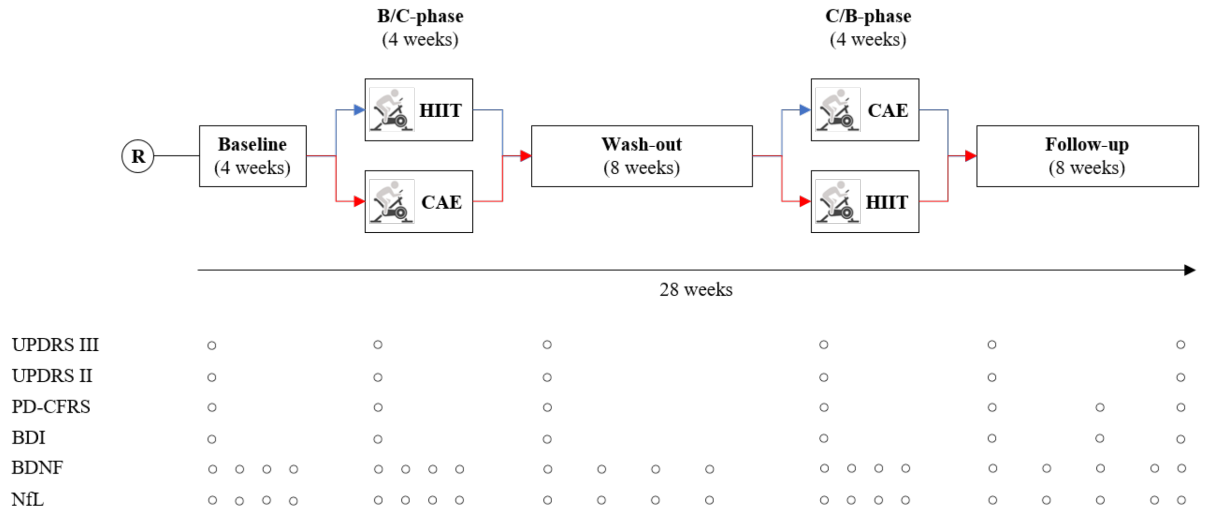

R=Randomization, HIIT=High Intensity Interval Training (B-phase), CAE=Continuous Aerobic Exercise (C-phase), UPDRS=Unified Parkinson's Disease Rating Scale (UPDRS II: motor experiences of daily living; UPDRS III: motor examination), PD-CFRS=Parkinson's Disease Cognitive Functional Rating Scale, BDI=Beck Depression Inventory, BDNF=Brain-Derived Neurotrophic Factor, NfL=Neurofilament Light. ○ represents the assessment schedule.

Supplementary Table S1 Change scores (absolute values or beta (β)) on secondary outcome measures of participant 1.

	**Baseline**	**CAE**	**Wash-out**	**HIIT**	**Follow-up**
SCWT (sec)					
• 1	-4.07	0.67	2.55/-5.28	3.84	-4.84/-1.29
• 2	0.77	1.61	0.79/-11.3	-5.42	-0.93/-0.83
• 3	-1.02	-0.31	-6.47/-4.92	5.46	-9.41/1.15
TMT (sec)					
• A	-14.33	-0.81	1.64/-6.64	-0.97	-2.64/0.98
• B	-16.89	2.53	21.17/-12.13	-12.59	3.34/4.02
NEAI (points)	2	-2	NA/3	NA	NA/NA
PDQ-39 (points)	-2	NA	1/1	-1	NA/4
SCOPA (points)					
• DS	NA	-1	1/-1	-3	2+1
• NS	1	-7	NA/NA	NA	NA/-1
10MWT (sec)	β=0.098	β=-0.203	β=0.028	β=-0.053	β=0.029
TUG (sec)	β=2e-15	β=-0.077	β=-0.148	β=0.01	β=-1e-15
OLS (sec)					
• RO	β=5.222	β=3.834	β=-1.901	β=-4.778	β=2.716
• RC	β=-0.338	β=0.205	β=-0.192	β=0.187	β=0.152
• LO	β=8.75	β=6.391	β=-2.542	β=2.847	β=-5.981
• LC	β=-0.166	β=0.278	β=0.266	β=-0.523	β=0.017
VAS (points)					
• Cognition	β=0.254	β=-0.261	β=-0.067	β=0.128	β=0.099
• Mood	β=0.05	β=-0.516	β=0.1597	β=0.103	β=-0.1286
• Sleep	β=0.234	β=0.216	β=0.0713	β=0.442	β=-0.2262
Physical inactivity (min)	β=-19.93	β=0.24	β=8.04	β=-0.58	β=22.18

SCWT=Stroop Color Word Test, TMT=Trail Making Test, NEAI=Nottingham Extended ADL Index, PDQ-39=Parkinson's Disease Questionnaire (39 items), SCOPA=Scales for Outcomes in Parkinson's disease, DS=daytime sleepiness, NS=nighttime sleep problems, 10MWT=10-Meter Walk Test, TUG,=Timed Up-and-Go, OLS=One Leg Stance, RO=right leg/eyes open, RC=right leg/eyes closed, LO=left leg/eyes open, LC=left leg/eyes closed, VAS=Visual Analogue Scale, sec=seconds, min=minutes, NA=not applicable.

Supplementary Table S2 Change scores (absolute values or beta (β)) on secondary outcome measures of participant 2.

	**Baseline**	**CAE**	**Wash-out**	**HIIT**	**Follow-up**
SCWT (sec)					
• 1	2.71	-2.11	-0.48/-1.66	-1.26	-1.25/9.62
• 2	-2.87	5.71	-4.92/2.9	3.22	1.66/1.43
• 3	-8.13	26.88	-5.01/4.36	-6.6	-16.92/4.85
TMT (sec)					
• A	-6.8	0.98	-13.35/17.31	1.98	3.42/-17.54
• B	-13.01	69.4	-57.81/-17.16	-47.12	3.07/79.63
NEAI (points)	6	NA	NA/ NA	NA	NA/2
PDQ-39 (points)	-1	NA	-2/2	-1	1/2
SCOPA (points)					
• DS	-1	NA	NA/NA	NA	1/-1
• NS	NA	NA	NA/-1	NA	NA/NA
10MWT (sec)	β=-0.204	β=0.1	β=0.056	β=-0.007	β=0.276
TUG (sec)	β=-0.341	β=0.123	β=0.011	β=0.067	β=-0.06
OLS (sec)					
• RO	β=1.431	β=5.215	β=-2.039	β=2.847	β=0.45
• RC	β=-0.154	β=-0.041	β=-0.18	β=0.454	β=-0.131
• LO	β=2.486	β=-0.491	β=0.533	β=0.644	β=-0.566
• LC	β=-0.14	β=0.069	β=-0.161	β=0.635	β=-0.089
VAS (points)					
• Cognition	β=-0.19	β=0.34	β=-0.0072	β=0.109	β=0.0214
• Mood	β=-0.274	β=0.288	β=-0.143	β=0.202	β=-0.181
• Sleep	β=-0.664	β=0.297	β=0.0783	β=0.168	β=-0.05
Physical inactivity (min)	β=-35.88	β=18.44	β=-3.12	β=9.48	β=-1.66

SCWT=Stroop Color Word Test, TMT=Trail Making Test, NEAI=Nottingham Extended ADL Index, PDQ-39=Parkinson's Disease Questionnaire (39 items), SCOPA=Scales for Outcomes in Parkinson's disease, DS=daytime sleepiness, NS=nighttime sleep problems, 10MWT=10-Meter Walk Test, TUG,=Timed Up-and-Go, OLS=One Leg Stance, RO=right leg/eyes open, RC=right leg/eyes closed, LO=left leg/eyes open, LC=left leg/eyes closed, VAS=Visual Analogue Scale, sec=seconds, min=minutes, NA=not applicable.

Supplementary Table S3 Change scores (absolute values or beta (β)) on secondary outcome measures of participant 3.

	Baseline	HIIT	Wash-out
SCWT (sec)			
• 1	3.84	-6.51	0.61
• 2	-3.21	-1.57	-7.21
• 3	-8.06	-12.67	5.68
TMT (sec)			
• A	-0.43	4.51	-0.79
• B	32.6	-63.24	45.63
NEAI (points)	7	2	-8
PDQ-39 (points)	1	-1	2
SCOPA (points)			
• DS	NA	NA	1
• NS	1	-2	NA
10MWT (sec)	β=-0.133	β=-0.435	β=-0.055
TUG (sec)	β=0.176	β=0.256	β=0.72
OLS (sec)			
• RO	β=10.528	β=-8.495	β=3.145
• RC	β=-0.296	β=-0.094	β=3.425
• LO	β=2.833	β=-1.226	β=1.01
• LC	β=-0.149	β=-0.038	β=0.005
VAS (points)			
• Cognition	β=-0.134	β=0.001	β=-0.0714
• Mood	β=0.064	β=0.076	β=-0.063
• Sleep	β=0.124	β=0.386	β=0.1976
Physical inactivity (min)	-	-	-

SCWT=Stroop Color Word Test, TMT=Trail Making Test, NEAI=Nottingham Extended ADL Index, PDQ-39=Parkinson's Disease Questionnaire (39 items), SCOPA=Scales for Outcomes in Parkinson's disease, DS=daytime sleepiness, NS=nighttime sleep problems, 10MWT=10-Meter Walk Test, TUG,=Timed Up-and-Go, OLS=One Leg Stance, RO=right leg/eyes open, RC=right leg/eyes closed, LO=left leg/eyes open, LC=left leg/eyes closed, VAS=Visual Analogue Scale, sec=seconds, min=minutes, NA=not applicable.

Supplementary Table S4 Changes (improvement or worsening) on outcome measures according to the smallest detectable change, per study phase

	Baseline	CAE	Wash-out		HIIT	Follow-up	
			Week 9 to 13	Week 13 to 17		Week 21 to 25	Week 25 to 28
UPDRS III	1/3 worsened	2/2 improved	N/A	2/2 worsened	2/3 improved	N/A	
UPDRS II	1/3 improved	1/2 improved	1/3[2] worsened	1/2 worsened	2/3 improved	1/2 worsened	
PD-CFRS	1/2[1] worsened	NA	NA	NA	1/2[1] worsened	1/2[1] improved	NA
	1/2[1] improved						
BDI	1/3 improved	1/2 improved	2/3 worsened	1/2 improved	1/2[1] worsened	1/2 improved	1/2 worsened
	1/3 worsened		1/3[2] improved		1/2[1] improved	1/2 worsened	1/2 improved
BDNF	2/3 worsened	1/2 worsened	2/2 improved		2/3 improved	2/2 improved	
	1/3 improved	1/2 improved			1/3 worsened		
NfL	1/3 worsened	1/2 worsened	2/2 improved		1/3 worsened	1/2 improved	
	2/3 improved	1/2 improved			2/3 improved	1/2 worsened	

UPDRS III=Unified Parkinson's Disease Rating Scale – motor examination, UPDRS II=Unified Parkinson's Disease Rating Scale – motor experiences of daily living, PD-CFRS=Parkinson's Disease Cognitive Functional Rating Scale, BDI=Beck Depression Inventory, BDNF=Brain-Derived Neurotrophic Factor, NfL=Neurofilament Light, CAE=Continuous Aerobic Exercise, HIIT=High Intensity Interval Training.

[1]There was no improvement possible in the third participant because of a lowest score, representing no impairment, at the start of that study phase.

[2]Change in outcome measure after the HIIT intervention
